# Peer Review of “Using Structural Equation Modelling in Routine Clinical Data on Diabetes and Depression: Observational Cohort Study”

**DOI:** 10.2196/38007

**Published:** 2022-04-27

**Authors:** Chelsea Jones

**Affiliations:** 1 Faculty of Rehabilitation Medicine University of Alberta Edmonton, AB Canada

**Keywords:** depression, diabetes, electronic health records, acute care, PLS-SEM, path analysis, equation modelling, accident, emergency care, emergency, structural equation modelling, clinical data

*This is a peer-review report submitted for the paper “Using Structural Equation Modelling in Routine Clinical Data on Diabetes and Depression: Observational Cohort Study.”*

## Round 1 Review

### General Comments

This paper [1] takes structural equation modelling (SEM) and uses it in a novel way that could be beneficial for researchers and clinicians alike. The results and discussion are transparent, and do not overstate the findings. The researchers created a complex model that could demonstrate the benefits of use of this data analysis method in other health care contexts. The future directions and recommendations are realistic.

### Specific Comments

#### Major Comments

1. Lacks a statement of the study design. SEM is the method of analysis, not the study design.

#### Minor Comments

1. Write out “A&E” in title and first mention in text of abstract.
2. In the Introduction and second section, you have 2 statements that are in close proximity and convey similar information. I would consider revising. Introduction statement: “Therefore, we sought to determine whether SEM could be used to make this data set more ‘research friendly’ by attempting to create clinical constructs and model some well-known clinical associations between depression and accident & emergency (A&E) use in patients with type 2 diabetes.” Next section statement: “Therefore, we sought to test whether SEM could be applied to a large routine clinical data set from East London to model these associations between depression, diabetic care, diabetic control, and A&E utilization, while assessing the impact of current mental health care provision.” Perhaps go with the second one.
3. Measures of Mental Health Diagnosis and Care - The information on the AUDIT seems misplaced or excessive since other outcome measures are not explained in that amount of detail. Consider removing: “Scores on the AUDIT range from 0-40, with higher scores indicating higher risk of dependence. The AUDIT C consists of the three consumption questions from the AUDIT and scores can range from 0-12, with higher scores indicating higher risk.”
4. I don't think you need to state this: “A full description of the adult mental health care cluster codes used by the NHS can be found here: (link).” Just state those are the clusters you chose, and why.
5. Data Source: Consider explaining what the intended purpose of each data source/database is. These are largely unknown to anyone outside the UK health care context and will require more detail.
6. More explanation of what partial least squares SEM (PLS-SEM) is might be beneficial for the reader.
7. May benefit from explanation of why PLS versus covariance-based (CB) and other SEM types since the sample size was large (PLS-SEM is a great choice in my mind, but others may want more justification).
8. State whether the structural model is reflexive or formative and justification for this.
9. Discussion: there are 2 similar comments in close proximity: “This might be related to a problem with the data set, which will be described later in the Discussion” and “This is not in agreement with previous research, which has shown that improvement of depressive symptoms through the use of psychotherapy and pharmacotherapy is associated with improved glycemic control. The opposite association reported in this study is likely related to issues with data quality, which will be outlined later.”
10. In the Limitations section, link those statements to the above issue (10) for clarity.
11. A statement in Future Directions and Recommendations could address issues with the data set and what should/could be done to improve this.

## Abbreviations

CB-SEM: covariance-based structural equation modelling
PLS-SEM: partial least squares structural equation modelling
SEM: structural equation modelling
